# Can we mitigate the psychological impacts of social isolation using behavioural activation? Long-term results of the UK BASIL urgent public health COVID-19 pilot randomised controlled trial and living systematic review

**DOI:** 10.1136/ebmental-2022-300530

**Published:** 2022-10-12

**Authors:** Elizabeth Littlewood, Dean McMillan, Carolyn Chew Graham, Della Bailey, Samantha Gascoyne, Claire Sloane, Lauren Burke, Peter Coventry, Suzanne Crosland, Caroline Fairhurst, Andrew Henry, Catherine Hewitt, Kalpita Baird, Eloise Ryde, Leanne Shearsmith, Gemma Traviss-Turner, Rebecca Woodhouse, Judith Webster, Nick Meader, Rachel Churchill, Elizabeth Eddy, Paul Heron, Nisha Hicklin, Roz Shafran, Osvaldo Almeida, Andrew Clegg, Tom Gentry, Andrew Hill, Karina Lovell, Sarah Dexter-Smith, David Ekers, Simon Gilbody

**Affiliations:** 1 Health Sciences, University of York, York, UK; 2 Centre for Health and Population Science, Hull York Medical School, Hull, UK; 3 School of Medicine, Keele University, Keele, UK; 4 York Environmental Sustainability Institute, University of York, York, UK; 5 Research and Development Unit, Tees Esk and Wear Valleys NHS Foundation Trust, Darlington, UK; 6 Faculty of Medicine and Health, University of Leeds, Leeds, UK; 7 Faculty of Medical Sciences, University of Newcastle, Newcastle upon Tyne, UK; 8 Cochrane Common Mental Disorders Group, University of York, York, UK; 9 Faculty of Health and Life Sciences, University of Liverpool, Liverpool, UK; 10 Department of Psychology, Royal Holloway University of London, Egham, UK; 11 PPP, University College London Institute of Child Health, London, UK; 12 Paediatric Psychology Services, Great Ormond Street Hospital for Children NHS Foundation Trust, London, UK; 13 UWA Medical School, The University of Western Australia, Perth, Western Australia, Australia; 14 Bradford Institute for Health Research, Bradford Royal Infirmary, Bradford, UK; 15 Health and Care Policy, Age UK, London, UK; 16 Division of Nursing, Midwifery and Social Work, The University of Manchester, Manchester, UK; 17 Centre for Health and Population Sciences, Hull York Medical School, York, UK

**Keywords:** Adult psychiatry, Depression & mood disorders

## Abstract

**Background:**

Behavioural and cognitive interventions remain credible approaches in addressing loneliness and depression. There was a need to rapidly generate and assimilate trial-based data during COVID-19.

**Objectives:**

We undertook a parallel pilot RCT of behavioural activation (a brief behavioural intervention) for depression and loneliness (Behavioural Activation in Social Isolation, the BASIL-C19 trial ISRCTN94091479). We also assimilate these data in a living systematic review (PROSPERO CRD42021298788) of cognitive and/or behavioural interventions.

**Methods:**

Participants (≥65 years) with long-term conditions were computer randomised to behavioural activation (n=47) versus care as usual (n=49). Primary outcome was PHQ-9. Secondary outcomes included loneliness (De Jong Scale). Data from the BASIL-C19 trial were included in a metanalysis of depression and loneliness.

**Findings:**

The 12 months adjusted mean difference for PHQ-9 was −0.70 (95% CI −2.61 to 1.20) and for loneliness was −0.39 (95% CI −1.43 to 0.65).

The BASIL-C19 living systematic review (12 trials) found short-term reductions in depression (standardised mean difference (SMD)=−0.31, 95% CI −0.51 to −0.11) and loneliness (SMD=−0.48, 95% CI −0.70 to −0.27). There were few long-term trials, but there was evidence of some benefit (loneliness SMD=−0.20, 95% CI −0.40 to −0.01; depression SMD=−0.20, 95% CI −0.47 to 0.07).

**Discussion:**

We delivered a pilot trial of a behavioural intervention targeting loneliness and depression; achieving long-term follow-up. Living meta-analysis provides strong evidence of short-term benefit for loneliness and depression for cognitive and/or behavioural approaches. A fully powered BASIL trial is underway.

**Clinical implications:**

Scalable behavioural and cognitive approaches should be considered as population-level strategies for depression and loneliness on the basis of a living systematic review.

**WHAT IS ALREADY KNOWN ON THIS TOPIC**

- Older people with long-term conditions have been impacted by COVID-19 pandemic restrictions and have experienced social isolation. In turn, this puts them at risk for depression and loneliness, and these are bad for health and well-being. Psychosocial approaches, such as behavioural activation, could be helpful.
- Trial-based evidence is needed to demonstrate if it is possible to address the onset, or mitigate the impact, of loneliness and depression.
- There are a few studies of brief psychosocial interventions to mitigate depression and loneliness, and it is important to know how emerging trial-based data adds to existing evidence.

**WHAT THIS STUDY ADDS**

- There was preliminary evidence that levels of loneliness were reduced at 3 months when behavioural activation was offered.
- At longer-term (12-month) follow-up, there was a potential positive impact.
- When Behavioural Activation in Social Isolation data were assimilated into a living systematic review there is clear evidence of impact of brief psychological interventions on depression and loneliness in the short term. More research into the longer-term impact is needed.

**HOW THIS STUDY MIGHT AFFECT RESEARCH, PRACTICE OR POLICY**

- Cognitive and/or behavioural interventions show evidence of benefit which will be useful for policy-makers in offering support to people who are socially isolated.
- This research knowledge will be useful once the COVID-19 pandemic has passed, since loneliness is common in older populations and effective scalable solutions will be needed to tackle this problem.
- As new trial-based data emerges, our living meta-analysis will be updated since this is an area of active research.

## Introduction

The mental health of the population deteriorated during the COVID-19 pandemic.^1^ Many people reported social isolation, and the incidence of depression and anxiety particularly increased for older people and those with medical vulnerabilities.^2^ A plausible mechanism for this deterioration was that COVID-19 restrictions led to disruption of daily routines, loss of social contact and heightened isolation and increased loneliness, which are each powerful precipitants of mental ill health.^3^

Social isolation, social disconnectedness, perceived isolation and loneliness are known to be linked to common mental health problems, such as depression in older people.^3 4^ Loneliness is a risk factor for depression and seems detrimental to physical health and life expectancy.^5^ It is recognised that strategies that, for instance, maintain social connectedness could be important in ensuring the mental health of older people,^6^ particularly during the pandemic^3^ and in the planning for postpandemic recovery.^7^

The need for research to mitigate the psychological impacts of COVID-19, particularly loneliness, was highlighted as a priority,^8^ and we responded by designing and delivering one of a small number of psychotherapy trials programmes.^9^

Behavioural activation (BA) is an evidence-based psychological treatment that explores how physical inactivity, avoidance and low mood are linked and result in a reduction of valued activity.^10^ Small scale trials of BA delivered to socially isolated older people have produced encouraging preliminary results,^11^ but there is not yet sufficient research evidence to support whole-scale adoption, or to inform the population response to COVID-19 or in planning for postpandemic recovery. We; therefore, adapted an ongoing work programme into the role of BA in multiple long-term conditions (LTCs) in early-2020 to answer the following overarching question: ‘Can we prevent or ameliorate depression and loneliness in older people with LTCs during isolation?’.

In this paper, we present the long-term (12-month) results of the BASIL-C19 trial (Behavioural Activation in Social Isolation): a pilot randomised controlled trial (RCT) of manualised BA, adapted specifically to be delivered at scale and remotely (via the telephone or video call) for older adults who became socially isolated as a consequence of COVID-19. The long-term (12-month outcomes) complement the already-published short-term (up to 3 months) outcomes of the BASIL-C19 trial.^12^ In the short-term BASIL-C19 results, we demonstrated our ability to recruit to a trial during COVID-19 and found a statistically significant effect in reducing levels of loneliness in a vulnerable older population.

Research into loneliness is a rapidly evolving area, and therefore, we present the short-term and long-term results of the BASIL-C19 trial alongside all available randomised data in a prospective evidence synthesis and cumulative meta-analysis. We adopted the method of a ‘living systematic review’ which is a form of evidence synthesis that is continually updated, incorporating relevant new evidence as it becomes available.^13^

Existing reviews in this area are conventional systematic reviews^14–16^ and will not incorporate new emerging evidence until their next update, which for most reviews is unplanned or does not happen and is not responsive to new emerging evidence. The adoption of living systematic reviews, as a method, was accelerated during the COVID-19 pandemic to facilitate the rapid assimilation and mobilisation of trial-based evidence as soon as it becomes available and is our chosen method of evidence synthesis.^17^

## Trial methods

### Study design and participants

The BASIL-C19 pilot RCT was the first and only mental health trial adopted by the National Institute for Health and Care Research (NIHR) Urgent Public Health (UPH) programme (adopted on 28 May 2020).^18^ The BASIL-C19 pilot was designed to provide key information on methods of recruitment and training for intervention practitioners (hereafter BASIL Support Workers (BSWs)). The trial was registered on 9 June 2020 (ISRCTN94091479) and participants were recruited between 23 June 2020 and 15 October 2020. Older adults with LTCs were identified as being a ‘high-risk group’ for loneliness and depression as a consequence of social isolation under COVID-19 restrictions. They were recruited from primary care registers in the North East of England. Eligible and consenting participants were randomised to receive either usual primary care (with signposting to resources to support mental health during COVID-19) from their general practice (GP) or BA intervention in addition to usual care. Methods, recruitment, intervention uptake, retention, experience of the BA intervention for our target population and acceptability of the intervention are described in full in the short-term results paper.^12^

Inclusion criteria: Based on the Academy of Medical Sciences definition of multimorbidity,^19^ we recruited older adults (65 years or over) with two or more physical LTCs on primary care registers in two GPs in the North East of England. Participants included those subject to English Government guidelines regarding COVID-19 self-isolation, social distancing and shielding as relevant to their health conditions and age (though this was not a requirement and these requirements changed during the study period).

Exclusion criteria: Older adults who had cognitive impairment (ascertained on clinical grounds by the GP), bipolar disorder/psychosis/psychotic symptoms, alcohol or drug dependence, in the palliative phase of illness, had active suicidal ideation, were currently receiving psychological therapy or were unable to speak or understand English.

Potentially eligible participants were telephoned and those who expressed an interest in the study were contacted by a member of the research team to determine eligibility, obtain consent and collect baseline data. Interested patients could also complete an online consent form or contact the study team directly.

### Randomisation, concealment of allocation and masking

Eligible and consenting participants were randomised 1:1 to BA intervention or usual care using simple randomisation via an automated computer data entry system, administered remotely by the York Trials Unit, University of York. Participants, GPs, study clinicians or BSWs were not blinded to treatment allocation. Outcome assessment was by self-report, and study researchers facilitating the telephone-based outcome assessment were blind to treatment allocation.

### Intervention (BA)

The intervention (BA within a collaborative care framework) has been described elsewhere^20^ and was adapted for the purposes of the BASIL-C19 trial. The main adaptation was the use of telephone delivery, and the use of functional equivalence to maintain social interactions.

BA pays particular attention to the function the behaviour holds for an individual and that reinforcement is determined functionally. An important consequence of this view is the idea of functional equivalence. A specific form of a behaviour may have served a particular function for a person. However, that behaviour may no longer be possible due to physical health problems or COVID-19 lockdown. In this situation, an aim of treatment was to identify a functionally equivalent behaviour that is different and therefore still possible despite physical changes or shielding, but which may serve the same function for a person.

Intervention participants were offered up to eight sessions over a period of 4–6 weeks delivered by trained BSWs, accompanied by a BASIL Behavioural Activation booklet.

Sessions were delivered by BSWs remotely via telephone or video call, according to participant preference. The first session was scheduled to last approximately 1 hour, with subsequent sessions lasting approximately 30 min.

#### Comparator (usual GP care with signposting)

Participants in the control group received usual care as provided by their current National Health Service (NHS) and/or third sector providers. In addition, control participants were ‘signposted’ to reputable sources of self-help and information, including advice on how to keep mentally and physically well (eg, Public Health England ‘Guidance for the public on the mental health and well-being aspects of COVID-19’^21^ and Age UK.^22^

### Outcome measures

Demographic information obtained at baseline included: age, sex, LTC type, socioeconomic status, ethnicity, education, marital status and number of children.

The overarching aim of the BASIL-C19 pilot trial was to test the feasibility of the intervention and the methods of recruitment, randomisation and follow-up.^23^ The primary clinical outcome was self-reported symptoms of depression, assessed by the Patient Health Questionnaire-12 (PHQ-9),^24^ where higher scores indicate greater levels of depressive symptomatology. The PHQ-9 was administered at baseline, 1, 3 and 12 months postrandomisation by research staff blind to treatment allocation. Other secondary clinical outcomes measured at baseline, 1, 3 and 12 months were health-related quality of life (Short Form-12v2 (SF-12v2) Mental Component Scale (MCS) and Physical Component Scale (PCS)),^25^ Generalised Anxiety Disorder-7 (GAD-7),^26^ perceived social and emotional loneliness (De Jong Gierveld Scale—11 items loneliness scale) and questions relating to COVID-19 circumstances and adherence to government guidelines.^27^ Findings from 1-month to and 3-month outcomes have been presented elsewhere,^12^ along with information on intervention compliance.

### Sample size and statistical analysis

#### Sample size

Sample size calculations were based on estimating attrition and SD of the primary outcome. We aimed to recruit 100 participants. The intervention was delivered by BSWs and allowed for potential clustering by BSWs assuming an intracluster correlation of 0.01 and mean cluster size of 15 based on previous studies.^20^ The effective sample size was therefore 88. Anticipating 15%–20% of participants would be lost to follow-up (17% in the CASPER trial of older adults,^20^ this would result in an effective sample size of at least 70 participants, which is sufficient to allow reasonably robust estimates of the SD of the primary outcome measure to inform the sample size calculation for a definitive trial.^28^

#### Statistical analysis

**Figure 1:**
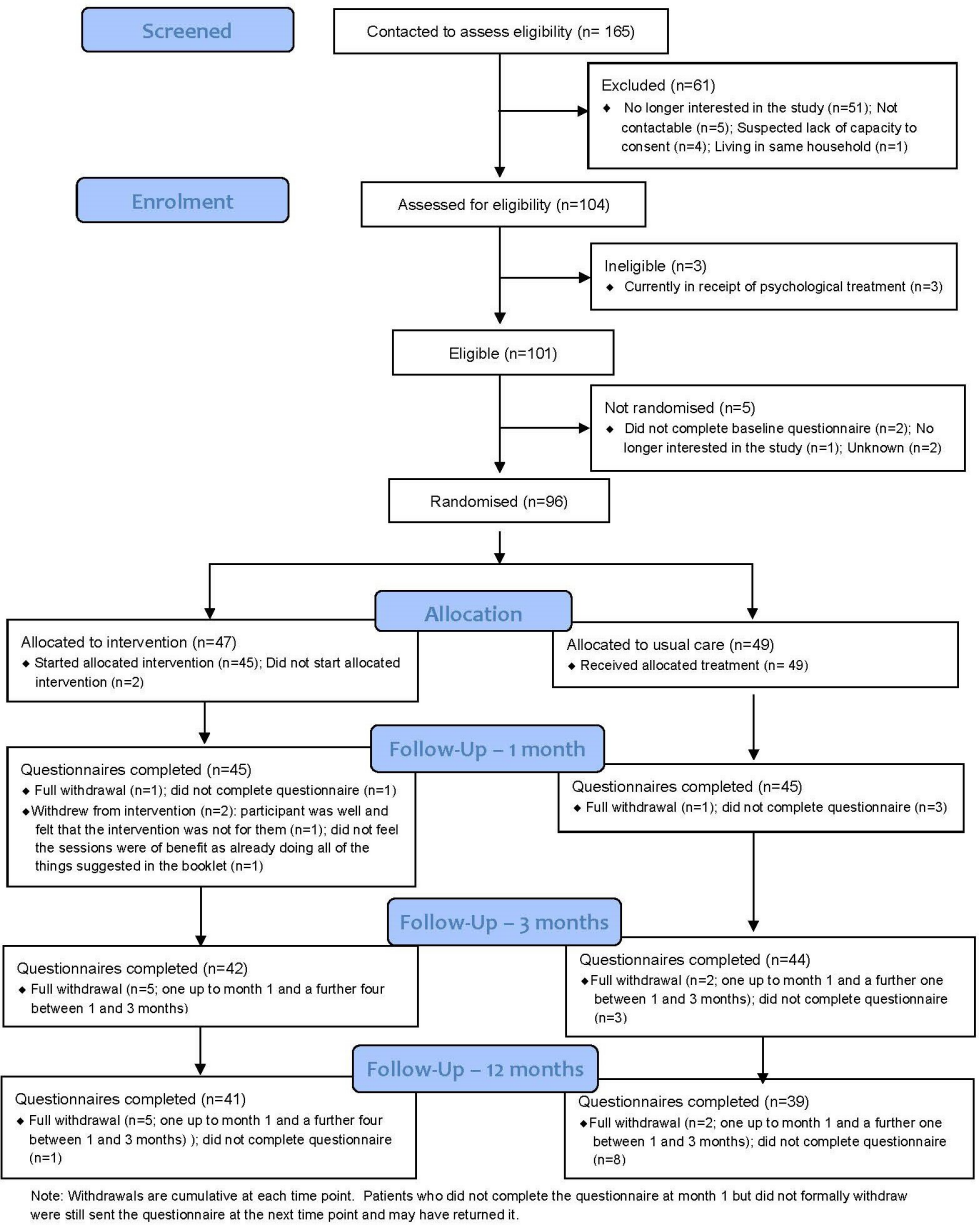
Basil CONSORT flow diagram. CONSORT, Consolidated Standards of Reporting Trials.

This study is reported as per the Consolidated Standards of Reporting Trials (CONSORT) guideline. The flow of participants through the pilot trial is shown in a CONSORT flow diagram (figure 1). Differences in the clinical outcomes between the two groups were compared at 12 months. This was done using a covariance pattern, mixed-effect linear regression model incorporating all postrandomisation time points. Treatment group, time point, a treatment-by-time interaction and the baseline score of the outcome of interest were included as fixed effects, and participant as a random effect (to account for the repeated observations per participant).

Different covariance structures were applied to the model. An unstructured covariance pattern for the correlation between the observations for a participant over time was specified in the final model based on Akaike’s information criterion (smaller value preferred).

An estimate of the difference between treatment groups in all outcome measures was extracted from the models for the 12-month time point, and overall, with a 95% CI as preliminary estimates of effect, but this pilot trial was not powered to show efficacy. Model assumptions were checked as follows: the normality of the standardised residuals was visually assessed using a QQ plot, and homoscedasticity by means of a scatter plot of the standardised residuals against fitted values. No concerning deviations were noted.

### Prospective meta-analysis of trial-based data

Using all available trial data to February 2022, we incorporated studies from an earlier Cochrane^16^ and non-Cochrane^15^ meta-analyses of cognitive and/or behavioural interventions to prevent or mitigate loneliness and depression in adult populations in light of the BASIL-C19 results. The planned living meta-analysis protocol was registered on the PROSPERO database (review protocol CRD42021298788).

We searched PubMed, EMBASE, PsycINFO from inception to February 2022 using the MetaPsy database, and also scrutinised the bibliography of two recent systematic reviews in this area to identify additional studies (a Cochrane review^16^ and a 2021 systematic review^15^ by the current authors). Eligible interventions included first, second or third wave cognitive–behavioural therapies (CBT) seeking to improve or prevent loneliness, as well as other CBT interventions where the focus is on improving common mental health problems but in which loneliness or a related construct is measured as an outcome. We studied depression and/or loneliness as the main outcomes of interest, under the advice of the BASIL Lived Experience Advisory Panel. We calculated a standardised mean difference (SMD) with 95% CI. SMD represents the size of the intervention effect of each study compared with the between-participant variability in outcome measurements recorded in each individual study. We categorised the postintervention outcomes into short-term outcomes (<6 months, including end of treatment time points), medium-term (≥6 to <12 months) and long-term outcomes (≥12 months). If a study reported follow-up outcomes at more than one time point within one of these time frames, we selected the outcome at the latest point within the time frame. We conducted a random effects meta-analysis, and included the BASIL-C19 study evidence. We tested for small study bias using Egger’s approach and test.^29^

## Results

### Participant recruitment, characteristics and follow-up

Ninety-six participants were randomised (47 to the BA intervention group; and 49 to usual care with signposting group), of which 80 (83.3%) completed the 12-month follow-up and valid scores were available for 79 (82.3%) (see figure 1).

The mean age of randomised participants was 74 years (SD 5.5) and most were white (n=92, 95.8%). Nearly two-thirds of the sample were female (n=59, 61.5%) (table 1), and the most common long-term health problems were cardiovascular conditions. Mean depression scores were indicative of mild depression (BA mean=7.5, SD 6.2; usual care mean=6.0, SD 5.6). There was reasonable balance in baseline characteristics at randomisation between the two groups.

**Table 1:** Unadjusted and adjusted mean differences between the BA and usual care groups by time point

Mean difference (95% CI)	1 month		3 month		12 month		Over 12 months
Outcome	Unadjusted	Adjusted*	Unadjusted	Adjusted*	Unadjusted	Adjusted*	Adjusted*
Patient Health Questionnaire-9 (primary outcome)	−1.44 (−3.66 to 0.77)	−0.50 (−2.01 to 1.01)	−0.39 (−2.70 to 1.91)	0.19 (−1.36 to 1.75)	−0.59 (−2.92 to 1.74)	−0.70 (−2.61 to 1.20)	−0.41 (−1.65 to 0.83)
Generalised Anxiety Disorder-7	−0.54 (−2.52 to 1.44)	0.20 (−1.33 to 1.73)	−0.16 (−2.09 to 1.78)	0.31 (−1.08 to 1.70)	−0.97 (−2.93 to 0.99)	−0.67 (−2.31 to 0.97)	−0.18 (−1.35 to 0.98)
De Jong Gierveld scale (total)	0.13 (−1.14 to 1.41)	0.28 (−0.51 to 1.06)	−0.86 (−2.14 to 0.43)	−0.87 (−1.56,–0.18)	0.07 (−1.31 to 1.45)	−0.39 (−1.43 to 0.65)	−0.32 (−0.97 to 0.34)
De Jong Gierveld Emotional Loneliness Subscale	0.07 (−0.68 to 0.81)	0.14 (−0.39 to 0.67)	−0.36 (−1.09 to 0.36)	−0.37 (−0.85 to 0.11)	0.19 (−0.70 to 1.08)	−0.05 (−0.74 to 0.65)	−0.16 (−0.57 to 0.26)
De Jong Gierveld Social Loneliness Subscale	0.07 (−0.68 to 0.81)	0.14 (−0.42 to 0.69)	−0.50 (−1.22,–0.23)	−0.50 (−1.00,–0.01)	−0.12 (−0.84 to 0.60)	−0.33 (−0.88 to 0.22)	−0.14 (−0.55 to 0.26)
Short Form-12v2 (Physical Component Score)†	1.40 (−3.42 to 6.22)	0.34 (−4.17 to 4.85)	0.81 (−4.16 to 5.77)	0.11 (−4.46 to 4.67)	−0.04 (−5.39 to 5.30)	−0.53 (−4.15 to 3.09)	−0.27 (−2.73 to 2.18)
Short Form-12v2 (Mental Component Score)†	3.60 (−1.17 to 8.37)	1.91 (−2.64 to 5.15)	2.09 (−2.48 to 6.65)	1.26 (−2.64 to 5.15)	2.17 (−2.54 to 6.89)	3.61 (−0.22 to 7.44)	3.22 (0.22 to 6.21)

*Adjusted for the baseline score of the outcome.

†Positive difference indicates better health in intervention group.

GAD-7, Generalised Anxiety Disorder 7; PHQ-9, Patient Health Questionnaire 9; SF-12, Short Form 12.

### Outcome data and between-group comparisons at 12 months

Eighty randomised participants (83.3%) completed the 12-month follow-up and valid primary and secondary outcome data were available for 79 (82.3%) participants (one participant commenced the questionnaire but then felt too unwell to continue and did not complete any of the outcome measures). At 12 months, unadjusted between-group mean differences was in the direction of the intervention for the Patient Health Questionnaire-9 (PHQ-9), Generalised Anxiety Disorder-7 (GAD-7), De Jong Social Loneliness and the Short Form-12 (SF-12) Mental Component Score (MCS), and usual care for De Jong total and the Emotional Loneliness subscale, and the Short Form-12 (SF-12) Physical Component Score (PCS). The point estimate adjusted mean difference between groups in the PHQ-9 indicated lower severity in the intervention group at 12 months (−0.70, 95% CI −2.61 to 1.20), with an overall difference of −0.41 (95% CI −1.65 to 0.83) across all time points. The width of confidence intervals included benefit, harm and no overall effect. The adjusted mean difference for the total De Jong Gierveld score indicated lower severity in the intervention group at 12 months (−0.39, 95% CI −1.43 to 0.65), with an overall difference of −0.32 (95% CI −0.97 to 0.34) across all time points. The direction of effect in long-term follow-up was consistent, though the majority were non-significant (table 1) and the width of CIs included benefit, harm and no overall effect. For mental health-related quality of life (the SF12 mental component score), there was an overall benefit across all time points (3.22, 95% CI 0.22 to 6.21). There were no adverse events attributed to the trial intervention or participation in the pilot trial.

### Living systematic review, incorporating BASIL-C19 data with all available trials data

We identified 12 studies (including BASIL-C19) that evaluated cognitive or behavioural interventions and reported either loneliness or depression outcomes (or both) (Gilbody-BASIL 2021,^12^ Choi- Pepin 2021,^11 30^ Kall 2020,^31 32^ Kall 2021,^33^ Soucy 2019,^34^ Williams 2004,^35^ Zhang 2018,^36^ Cohen-Mansfield 2018,^37^ Cresswell 2012,^38^ Jarvis 2019,^39^ Theeke 2016^40^ and Almeida 2022).^41^ The details of these trails are summarised in online supplemental table 1.

10.1136/ebmental-2022-300530.supp1 Supplementary data

When we applied the Cochrane Risk of Bias (RoB) tool^42^ to the 12 included studies, all were judged at some RoB. For most individual RoB domains, the majority of studies were judged to have some concerns or a higher RoB. For the first domain, bias arising from the randomisation process, five studies were judged to have some concerns and one study to be at high risk. For the second domain, bias due to deviations from the intended protocol, the picture was more mixed, with five at low risk, five having some concerns and two at high risk. For the third domain, bias due to missing outcome data, just under half were judged at high risk and three had some concerns. For the fourth domain, bias in measurement of the outcome, the majority (seven studies) judged to be at high risk or to have some concerns. For the final domain, bias in selection of reported outcomes, majority (eight studies) were judged to have some concerns.

When we pooled data for cognitive and/or behavioural interventions, all twelve studies assessed loneliness in the short term (≥6 months) and there was strong evidence of benefit for cognitive and/or behavioural interventions (986 participants, SMD=−0.48, 95% CI −0.70 to −0.27, I^2^=64.3%). Four studies assessed loneliness in the long term (≥12 months) and there was some evidence of benefit (321 participants, SMD=−0.20, 95% CI −0.40 to −0.01, I^2^=0%). Nine studies assessed depression in the short term, and there was strong evidence of benefit (775 participants, SMD=−0.31, 95% CI −0.51 to −0.11, I^2^=38.0%). Four studies assessed depression in the long term, at 12+ months, and although favouring cognitive and/or behavioural interventions the 95% CI was wider due to fewer studies reporting at this time point (324 participants, SMD=−0.20, 95% CI −0.47 to 0.07, I^2^=35.7%). No studies reported medium term (≥6 to <12 month) data. In all analyses, the level of between-study heterogeneity was low to moderate.

There were sufficient short-term outcome data to allow subgroup analyses according to whether the intervention was a generic psychological therapy versus therapy that focuses specifically on loneliness. We were also able to compare the effects in working age adults compared with older adult populations. There were insufficient studies to allow us to compare the effects of purely behavioural intervention with those that focused on or included cognitive elements.

For loneliness as an outcome, we found that although the effect estimate was larger in working age adults (SMD −0.57, 95% CI −0.84 to −0.30, n=5 studies) than in studies in older adult populations (SMD −0.46, 95% CI −0.83 to −0.11, n=7 studies), differences between subgroups were not statistically significant (χ2=0.24, df=1, p=0.62). The effect estimate for loneliness was larger in studies using loneliness-specific intervention (SMD −0.61, 95% CI −0.87 to −0.34, No. trials=9) compared with interventions using generic interventions (SMD −0.19, 95% CI −0.45 to 0.08, No. trials=3) and the difference between subgroups was statistically significant (χ^2^=4.81, df=1, p=0.03).

For depression as an outcome, we found that the effect estimates were similar in working age adults (SMD −0.37, 95% CI −0.69 to −0.06, n=4 studies) compared with studies in older adult populations (SMD −0.26, 95% CI −0.55 to 0.03, n=5 studies) and differences between subgroups were not statistically significant (χ^2^=0.26, df=1, p=0.61). The effect estimate for depression was also larger in studies using loneliness-specific intervention (SMD −0.41, 95% CI −0.68 to −0.13, No. trials=6) compared with interventions using generic interventions (SMD −0.15, 95% CI −0.36 to 0.07, No. trials=3), but the difference between subgroups was not statistically significant (χ^2^=2.10, df=1, p=0.15).

Where it was possible to test for small study and publication bias, there was evidence of funnel plot asymmetry for short term loneliness (Egger’s test p<0.05), but not for short term depression (Egger’s test p=0.76).

**Figure 2:**
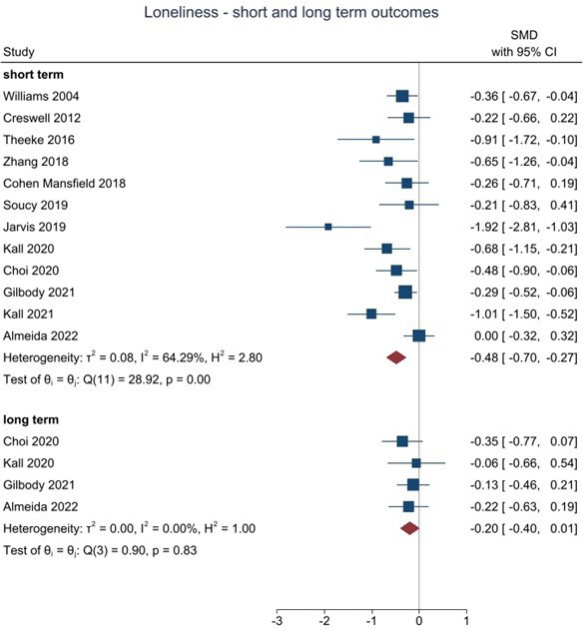
Living meta-analysis of behavioural and cognitive trials targeting loneliness in socially isolated populations. SMD, standardised mean difference.

**Figure 3:**
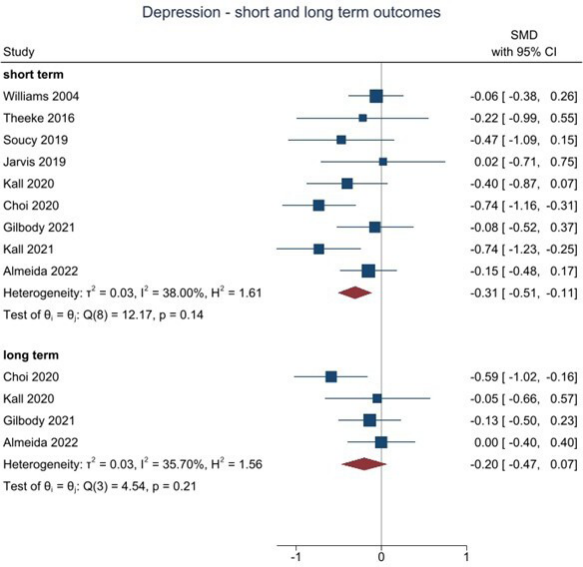
Living meta-analysis of behavioural and cognitive trials targeting depression in socially isolated populations. SMD, standardised mean difference.

## Discussion

The BASIL-C19 trial is an external pilot trial, designed to test an adapted behavioural intervention and to refine trial procedures before undertaking a full-scale trial. To our knowledge, this is one of only a small number of trials undertaken during COVID-19 to mitigate the psychological impact of the pandemic and its restrictions.^9^ We demonstrate that it was possible to trial a scalable intervention, and achieve good long-term follow-up rates under pandemic conditions. The pilot study was not deigned to have sufficient statistical power to test the effectiveness of BA and there are wide CIs. However, we were able to judge how the BASIL results add to existing trial-based evidence by undertaking a living systematic review.

We have previously reported the short-term outcomes where there was a statistically significant benefit in reducing loneliness,^12^ and here, we present the 12-month outcomes alongside a ‘living systematic review’, undertaken during the pandemic to evaluate accumulating evidence of cognitive and behavioural approaches in the prevention or mitigation of depression and loneliness. Our main meta-analytical finding is that the BASIL-C19 pilot trial results add to a growing body of trial-based research (summarised in a living systematic review) that demonstrates that brief psychological interventions can potentially offer clinical benefit to address both depression and loneliness. We also demonstrate the relative absence of long-term follow-up data, but note that the BASIL-C19 trial is one of only four trials to assess longer-term outcomes.

Research to date has shown behavioural approaches to be highly effective in the treatment of depression among older people^10 20 43 44^ and the preliminary results of the BASIL-C19 trial support this approach under COVID-19 restrictions and in mitigating loneliness^45^ in an at-risk population. On this basis a fully powered trial was planned and has been justified.

Our pilot trial was also undertaken rapidly and during the COVID-19 pandemic in early 2020; the time elapsed between the onset of the pandemic and the recruitment of the first participant was less than 3 months. We chose to study the impact of a plausible psychosocial intervention to mitigate depression and loneliness in an at-risk population of older people with multiple LTCs. It is also important that interventions to tackle the higher rates of depression and loneliness in all age groups are also developed and evaluated.

The BASIL-C19 trial was not designed or powered to detect effectiveness, and a fully powered pragmatic trial (BASIL+, ISRCTN63034289), is now underway to test for robust effects in important secondary outcomes such as loneliness with the benefit of greater statistical precision.^46^ We note the potential impacts of small study size in making baseline imbalances more likely to be observed by chance alone. We were able to adjust for such differences in our planned statistical analysis, but some anomalous results emerged adding caution to the interpretation of between group differences. For example, CI for loneliness changed quite substantially in the adjusted compared with the unadjusted model. We assume this is due to the increase in power and precision caused by baseline adjustment for the outcome. However, we also note that this pattern was not observed at any other time point.

The COVID-19 pandemic prompted a number of studies to understand the impacts of COVID-19,^47^ but there have been very few studies to evaluate psychosocial interventions to mitigate psychological impact.^9^ A clinical priority and policy imperative is to identify a brief and scalable intervention to prevent and mitigate loneliness, particularly in older people.^48^ The BASIL trials programme (including the living systematic review) will be informative in improving the mental health of populations in socially isolated at-risk populations after the pandemic has passed.^7^

We also emphasise that we have used, for the first time, the technique of ‘living systematic review’ to describe the impact of cognitive and/or behavioural interventions in addressing depression and loneliness in the face of social isolation. This will be updated in line with future and emerging trial-based evidence. The use of this technique was accelerated in many domains of health during the COVID-19 pandemic,^13 17^ and here, we present novel results in relation to loneliness. The living systematic review demonstrates that there are now multiple small-scale trials of interventions for loneliness. The strong meta-analytical signal of effect in reducing loneliness in the short term should be interpreted with some caution, because there is a potential small study and methodological biases, and larger well-designed studies are needed. We also note the range of populations included in trials in terms of age and the specific treatment modality. The living systematic review demonstrated that psychological approaches are likely to be equally effective in older adult and working age adult populations. It was also demonstrated that interventions designed to specifically target loneliness are likely to be more effective than unmodified cognitive and/or behavioural approaches in reducing levels of loneliness. More trials will be needed to explore this further. Finally, the living systematic review highlighted common methodological concerns among trials of brief psychological therapies, including suboptimal randomisation methods and selective reporting of outcomes.

It is not clear on the basis of the living systematic review whether behavioural or cognitive approaches are equally effective, and more trials-based research is needed to understand this. The broader literature shows the equivalence, in terms of effectiveness, of behavioural vs cognitive treatment modalities in treating depression,^49^ and it is not yet clear on the basis of the BASIL living systematic review whether this also applies to loneliness. We anticipate that further updates of the living systematic review will allow this to be explored further and that there is now a large-scale trial of a behavioural approach in follow-up.^46^

## Data Availability

Data are available on reasonable request. The BASIL research collective is especially keen that the BASIL data contributes to prospective meta-analyses and individual patient data meta-analyses. Requests for data sharing will be considered by the independent trial steering and data monitoring committee. Full underlying (non-aggregated) data cannot be made publicly available since the ethics approval of this study does not cover openly publishing non-aggregated data.
