# Supplementary material for: Can we mitigate the psychological impacts of social isolation using behavioural activation? Long-term results of the UK BASIL urgent public health COVID-19 pilot randomised controlled trial and living systematic review
Source: Evid Based Ment Health. 2022 Oct 12;25(e1):e49–57. doi: 10.1136/ebmental-2022-300530 (PMC9811092; doi:10.1136/ebmental-2022-300530)
Supplement: Supplementary data [file ebmental-2022-300530supp001.pdf]

Supplementary table 1: Randomised controlled trials included in the Living Systematic Review of cognitive and/or behavioural interventions for loneliness or depression

**Study**	**Population**	**Intervention**	**Loneliness and depression outcomes**
Gilbody-BASIL 2021 [1]	Older adults (65 years or over) with two or more physical long-term conditions (LTCs) on primary care registers in two general practices in the North East of England. Participants included those subject to English Government guidelines regarding COVID-19 self-isolation, social distancing and shielding as relevant to their health conditions and age.<br>N=96	Behavioural activation delivered over telephone by support workers (after two days training). The main adaptation was the use of telephone delivery, and the use of functional equivalence to maintain social interactions.	Loneliness: De Jong Gierveld Scale - 11 items loneliness scale<br>Depression: Patient Health Questionnaire-9 item (PHQ-9)
Choi Pepin 2021 [2, 3]	Older adults (aged 50+ and 60+) in receipt of home delivered meals. Cognitively intact and no substance abuse. Self report of feeling lonely and ≥6 of the possible score range of 3-9 on the 3-item UCLA Loneliness Scale).<br>N=89	Tele-BA: a brief, structured behavioural approach that aims to increase and reinforce wellness promoting behaviours (e.g., engaging in meaningful life activities aligned with personal values) and to decrease depressive behaviours. Tele-BA had five sessions	Loneliness: 8-item Patient-Reported Outcomes Measurement Information System Social Isolation Scale (PROMIS-L)<br>Depression: Patient Health Questionnaire-9 item (PHQ-9)
Kall 2020, [4] [5]	Aged 18 years and above, experience suffering as a result of loneliness (as assessed by the participant), score above the reported mean of 40 on the UCLA-LS-3, able to speak, read and write Swedish, have access to a computer with an internet connection, and, if taking psychopharmaceutic medication, remain on a stable dose for the duration of the study. Psychiatric comorbidity was allowed as long as the participant reported that loneliness was their primary concern	Internet CBT: Internet-Based Cognitive Behaviour Therapy for Loneliness over 8 weeks Delivered by final-year students enrolled at the 5-year clinical psychologist programme; all students had at least 18 months of prior theoretical and practical experience of	Loneliness: UCLA-LS-3<br>Depression: Patient Health Questionnaire-9 item (PHQ-9)

	N=73	CBT and received group supervision by two licensed psychologists during both the assessment and treatment phase	
Kall 2021 [6]	Aged 18 years and above, experience suffering as a result of loneliness (as assessed by the participant), score above the reported mean of 40 on the UCLA-LS-3, able to speak, read and write Swedish, have access to a computer with an internet connection, and, if taking psychopharmaceutic medication, remain on a stable dose for the duration of the study. Psychiatric comorbidity was allowed as long as the participant reported that loneliness was their primary concern<br>N= 170	Internet CBT: Internet-Based Cognitive Behaviour Therapy for Loneliness over 8 weeks<br>Delivered by final-year students enrolled at the 5-year clinical psychologist programme; all students had at least 18 months of prior theoretical and practical experience of CBT and received group supervision by two licensed psychologists during both the assessment and treatment phase	Loneliness: UCLA Loneliness Scale<br>Depression: Patient Health Questionnaire-9 item (PHQ-9)
Soucy 2019 [7]	Aged 18-65 years, with mild to moderate depressive symptoms evaluated using the 'Mini International Neuropsychiatric Interview (M.I.N.I) and the Patient Health Questionnaire (PHQ-9)<br>N=56	Behavioural Activation for depression<br>Individuals to re-engage in activities considered enjoyable, promote feelings of pleasure and accomplishment.<br>Participants provided with self-help BA manual and complete exercises. Interventionist called participants once every two weeks to use behaviour change techniques (e.g. problem	Loneliness: Laval university loneliness scale<br>Depression: Patient Health Questionnaire-9 item (PHQ-9)

		solving and goal setting)	
Williams 2004 [8]	Young adults (Navy recruits) attending Boot Camp Survival Training, at risk of depression (18 and above on the Beck Depression Inventory or 30 and above on the Perceived Stress Scale)<br>N=801	CBT (BOOTSTRAP)<br>using the strategies for coping, sense of belonging, decreasing thought distortions, and stress management. Encouraged participants to gain a sense of belonging through positive interactions with ship mates.	Loneliness: UCLA Loneliness Scale (20 item)<br>Depression: Beck Depression Inventory-II
Zhang 2018 [9]	Chinese higher education students, aged 17-25 years, elevated loneliness (one SD above mean population score), meditation naive, not taking psychiatric medication or receiving psychotherapy<br>N=59	Mindfulness based CBT: Taught theories, practiced mindfulness exercises, and discussed home practice. Based on the MBCT manual, substituting depression for loneliness	Loneliness: Indigenous loneliness scale<br>Depression: not measured
Cohen-Mansfield 2018 [10]	Aged 65 and above, feeling lonely (moderate level and above) and frequency (several times per week and above) of loneliness, not participating in social activities and expressing at least moderate desire to have additional company, able to participate based on cognitive function (>22 on Mini Mental State Examination), and no significant depression (Geriatric Depression Scale)<br>N=68 randomised	CBT: Face-to-face individual and/or group sessions<br>Individual sessions to identify and address individual personal barriers to social integration, discussion for social contacts and techniques, and use of local resources. Group sessions involved social skills practice and discussion on addressing barriers.	Loneliness: UCLA-8 loneliness scale<br>Depression: not measured
Cresswell 2012 [11]	Healthy older adult (aged 55-85 years), English-speaking, not currently participating in mind-body (e.g. meditation, yoga) more than once per week, non-smoker, mentally and physically	Mindfulness CBT for loneliness: Face-to-face group.<br>Trained mindfulness clinicians lead group sessions. All	Loneliness: UCLA-R Loneliness Scale (20 item)<br>Depression: not measured

	healthy for past three months, and not taking medications that affect immune, cardiovascular, endocrine,<br>or psychiatric functioning<br>N=40	maintained a daily mindfulness meditation practice	
Jarvis 2019 [12]	Older adults aged 60 years and above, willing to participate, cognitively intact on the Subjective Memory Complaint Clinical (SMCC), and either socially isolated (15 or above on the Friendship Scale),<br>lonely (2-6 on the de Jong Gierveld Loneliness Scale (DJGLS)) or with decreased mental wellbeing (13<br>or below on the World Health Organization-Five Wellbeing Index (WHO-5)).<br>N=32	CBT: Living in Network Connected Communities (mLINCC) CBT intervention to<br>reduce loneliness. Face-to-face group and individual sessions, and smartphone message via WhatsApp from CBT specialists.	Loneliness: De Jong Gierveld Loneliness scale<br>Depression: World Health Organisation-Five Wellbeing Index (WHO-5)
Theeke 2016 [13]	Aged 65 years or older, minimum loneliness score of 40 (revised 20-item UCLA Loneliness<br>scale), living in the community, diagnosed with at least one chronic illness, and voluntarily<br>signed an informed consent form<br>N=27	CBT LISTEN intervention over 5 weeks. A cognitive restructuring intervention for loneliness Taught sessions and writing activities on belonging, relationships, role in community, loneliness as a health challenge, and meaning of loneliness. Plus homework sessions. Face-to-face group intervention.	Loneliness: UCLA Loneliness Scale (20 item)<br>Depression: Geriatric Depression Scale (GDS)
Almeida 2022 [14]	Nursing home residents in Australia aged 60 years or over; reporting symptoms of low mood or hopelessness, or lack of interest or pleasure in usual activities. They also had to score 5 or more on the Patient Health Questionnaire (PHQ-9). Participants scored 18 or more on the Mini-Mental State Examination (MMSE), be able to communicate effectively in English, and have capacity to provide informed consent.<br>N=188	Behavioural Activation modified for nursing home residents: Package consisted of 5 to 7 modules comprising (1) understanding anxiety and depression, (2) anxiety and depression in older people, (3) promoting good mental health of older adults in the community, (4)	Loneliness: De Jong Gierveld Scale - 11 items loneliness scale<br>Depression: Patient Health Questionnaire-9 item (PHQ-9)

		promoting good mental health of aged care residents, (5) identifying and responding to suicidal ideation in aged care settings, (6) managing anxiety and depression of aged care residents, (7) looking after own mental health at work. Each module took about 25 minutes to complete	

References to included studies.

1. Gilbody S, Littlewood E, McMillan D, Chew-Graham CA, Bailey D, Gascoyne S, et al. Behavioural activation to prevent depression and loneliness among socially isolated older people with long-term conditions: The BASIL COVID-19 pilot randomised controlled trial. PLoS Med. 2021;18(10):e1003779. Epub 2021/10/13. doi: 10.1371/journal.pmed.1003779. PubMed PMID: 34637450; PubMed Central PMCID: PMCPMC8509874
2. Bruce ML, Pepin R, Marti CN, Stevens CJ, Choi NG. One Year Impact on Social Connectedness for Homebound Older Adults: Randomized Controlled Trial of Tele-delivered Behavioral Activation Versus Tele-delivered Friendly Visits. Am J Geriatr Psychiatry. 2021;29(8):771-6. Epub 2021/06/17. doi: 10.1016/j.jagp.2021.05.005. PubMed PMID: 34130906; PubMed Central PMCID: PMCPMC8286332.
3. Choi NG, Pepin R, Marti CN, Stevens CJ, Bruce ML. Improving Social Connectedness for Homebound Older Adults: Randomized Controlled Trial of Tele-Delivered Behavioral Activation Versus Tele-Delivered Friendly Visits. Am J Geriatr Psychiatry. 2020;28(7):698-708. Epub 2020/04/03. doi: 10.1016/j.jagp.2020.02.008. PubMed PMID: 32238297; PubMed Central PMCID: PMCPMC8767809.
4. Kall A, Jagholm S, Hesser H, Andersson F, Mathaldi A, Norkvist BT, et al. Internet-Based Cognitive Behavior Therapy for Loneliness: A Pilot Randomized Controlled Trial. Behav Ther. 2020;51(1):54-68. Epub 2020/02/02. doi: 10.1016/j.beth.2019.05.001. PubMed PMID: 32005340.
5. Käll A, Backlund U, Shafran R, Andersson G. Lonesome no more? A two-year follow-up of internet-administered cognitive behavioral therapy for loneliness. Internet interventions. 2020;19:100301.
6. Käll A, Bäck M, Welin C, Åman H, Bjerkander R, Wänman M, et al. Therapist-guided internet-based treatments for loneliness: a randomized controlled three-arm trial comparing cognitive behavioral therapy and interpersonal psychotherapy. Psychotherapy and Psychosomatics. 2021;90(5):351-8.
7. Soucy I, Provencher MD, Fortier M, McFadden T. Secondary outcomes of the guided self-help behavioral activation and physical activity for depression trial. J Ment Health. 2019;28(4):410-8. Epub 2018/05/04. doi: 10.1080/09638237.2018.1466031. PubMed PMID: 29722574.
8. Williams A, Hagerty BM, Yousha SM, Horrocks J, Hoyle KS, Liu D. Psychosocial effects of the BOOT STRAP intervention in Navy recruits. Mil Med. 2004;169(10):814-20. Epub 2004/11/10. doi: 10.7205/milmed.169.10.814. PubMed PMID: 15532347.

9. Zhang N, Fan Fm, Huang Sy, Rodriguez MA. Mindfulness training for loneliness among Chinese college students: A pilot randomized controlled trial. International Journal of Psychology. 2018;53(5):373-8.
10. Cohen-Mansfield J, Hazan H, Lerman Y, Shalom V, Birkenfeld S, Cohen R. Efficacy of the I-SOCIAL intervention for loneliness in old age: Lessons from a randomized controlled trial. J Psychiatr Res. 2018;99:69-75. Epub 2018/02/07. doi: 10.1016/j.jpsychires.2018.01.014. PubMed PMID: 29407289.
11. Creswell JD, Irwin MR, Burklund LJ, Lieberman MD, Arevalo JM, Ma J, et al. Mindfulness-Based Stress Reduction training reduces loneliness and pro-inflammatory gene expression in older adults: a small randomized controlled trial. Brain Behav Immun. 2012;26(7):1095-101. Epub 2012/07/24. doi: 10.1016/j.bbi.2012.07.006. PubMed PMID: 22820409; PubMed Central PMCID: PMCPMC3635809.
12. Jarvis MA, Padmanabhanunni A, Chipps J. An Evaluation of a Low-Intensity Cognitive Behavioral Therapy mHealth-Supported Intervention to Reduce Loneliness in Older People. Int J Environ Res Public Health. 2019;16(7):1305. Epub 2019/04/14. doi: 10.3390/ijerph16071305. PubMed PMID: 30979042; PubMed Central PMCID: PMCPMC6480633.
13. Theeke LA, Mallow JA, Moore J, McBurney A, Rellick S, VanGilder R. Effectiveness of LISTEN on loneliness, neuroimmunological stress response, psychosocial functioning, quality of life, and physical health measures of chronic illness. Int J Nurs Sci. 2016;3(3):242-51. Epub 2017/10/31. doi: 10.1016/j.ijnss.2016.08.004. PubMed PMID: 29082303; PubMed Central PMCID: PMCPMC5656260.
14. Almeida OP, Patel H, Velasquez D, Kelly R, Lai R, Ford AH, et al. Behavioural activation in nursing homes to treat depression (BAN-Dep): Results from a clustered, randomised, single-blinded, controlled clinical trial. The American Journal of Geriatric Psychiatry. 2022. doi: https://doi.org/10.1016/j.jagp.2022.05.009.
